# Managing Constipation in Adults With Cancer

**Published:** 2017-03-01

**Authors:** Rita J. Wickham

**Affiliations:** Rush University College of Nursing (Adjunct Faculty), Chicago, Illinois

## Abstract

Constipation is common in individuals with cancer, occurring in almost 60% of patients overall. The incidence increases in patients with advanced disease, particularly in those receiving opioid analgesics or medications with anticholinergic properties. Constipation is not uniformly assessed and therefore not recognized and appropriately managed in many instances. This can increase patients’ physical and psychological distress. Furthermore, there is scant research to support current management strategies for constipation. The objectives of this review are to explore the incidence of and risk factors for constipation in patients with cancer, to discuss the extent of the problem, to explore the nonpharmacologic and pharmacologic measures for constipation and fecal impaction, and to synthesize a laxative management. An extensive review of medical, pharmacy, and nursing literature was done to explore the physiology and pathogenesis of constipation; detail the mechanisms of action, onset of effect, approximate costs, and adverse effects of drugs for constipation; and condense clinical expert consensus recommendations for constipation, particularly in patients with cancer. Advanced practitioners (APs) and other clinicians play crucial roles in identifying individuals at risk for and experiencing constipation to help them use effective regimens, including over-the-counter laxatives, and perhaps adjunctive nondrug measures. Clinicians and patients must develop an agreed-upon language for identifying the severity and effects of constipation. In addition, both should understand which laxatives are most appropriate and which should be avoided for particular patients. Two prescription agents are also available, and understanding when they should be used is important for APs.

Constipation is a common and distressing problem for many individuals with cancer during treatment and palliative care, and perhaps even during survivorship; in too many instances, it goes unrecognized and untreated (McMillan, Tofthagen, Small, Karver, & Craig, 2013). Constipation can range from an annoying discomfort to life-threatening impaction with circulatory, cardiac, or respiratory symptoms (Clemens, Faust, Jaspers, & Mikus, 2013). This article will review the incidence, risk factors, assessment, and management of constipation in persons with cancer.

## NORMAL BOWEL HABITS AND CONSTIPATION

The range of normal bowel movements (BMs) in healthy people is arbitrarily defined as three BMs per day to three per week (Candy et al., 2015). In general, constipation occurs because prolonged bowel transit allows more water to be absorbed from feces through the bowel wall, which leads to hard, dry, and difficult-to-pass stools (Twycross, Sykes, Mihalyo, & Wilcock, 2012). Many authors use the Rome III criteria (Table 1) to define constipation characteristics, but these criteria for functional constipation do not consistently fit with constipation in advanced illness (Longstreth et al., 2006).

**Table 1:**
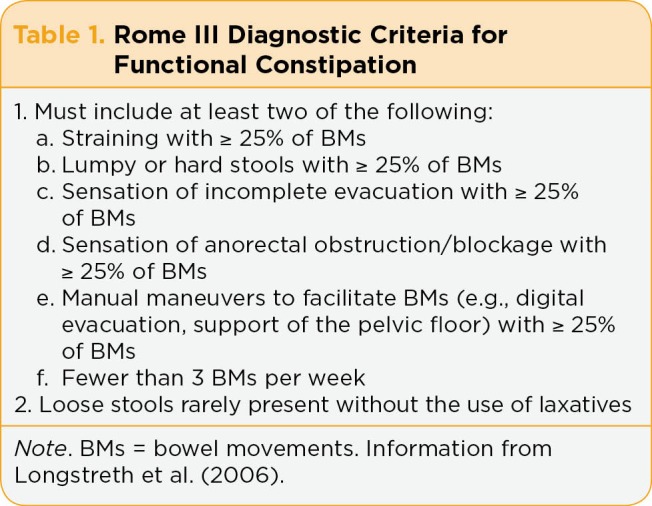
Rome III Diagnostic Criteria for Functional Constipation

Constipated stools can range from small, hard "rocks" to large bulky masses and may be accompanied by discomfort or pain (Clemens et al., 2013; Costilla & Foxx-Orenstein, 2014). Other related manifestations may include abdominal distention and bloating, urinary retention, nausea, anorexia, and rectal problems (e.g., hemorrhoids and anal fissures; Clemens et al., 2013). Constipation can also cause paradoxical or overflow diarrhea, as liquid stool passes around the obstructing constipated stool. Chronic constipation can also lead to fecal impaction, particularly in patients with advanced disease who have poor oral intake with little dietary fiber, dehydration, limited physical activity or immobility, or abdominal tumor (Hussain, Whitehead, & Lacy, 2014).

A total of 43% to 58% of patients with cancer report constipation (McMillan et al., 2013)—the third most common symptom (after pain and anorexia) in those with advanced disease (Clemens et al., 2013). In terminally ill patients, bowel dysfunction may occur in ≥ 80% of patients and in 90% of patients taking opioids (Downing, Kuziemsky, Lesperance, Lau, & Syme, 2007; Rhondali et al., 2013). Furthermore, drugs that contribute to an "anticholinergic load" are strongly implicated in constipation in palliative care patients (Clark, Lam, Agar, Chye, & Currow, 2010). As can be gleaned from Table 2 on the following page, constipation in patients with cancer is typically multicausal and related to organic, functional, and drug-related effects (Bharucha, Pemberton, & Locke, 2013; Clemens et al., 2013; Costilla & Foxx-Orenstein, 2014; Solomon & Cherny, 2006). For example, a patient’s constipation might be related to polypharmacy (taking an opioid analgesic for pain along with other drugs that have anticholinergic properties) and may be exacerbated by low physical activity, decreased oral intake of food and fluids, and diabetes.

**Table 2:**
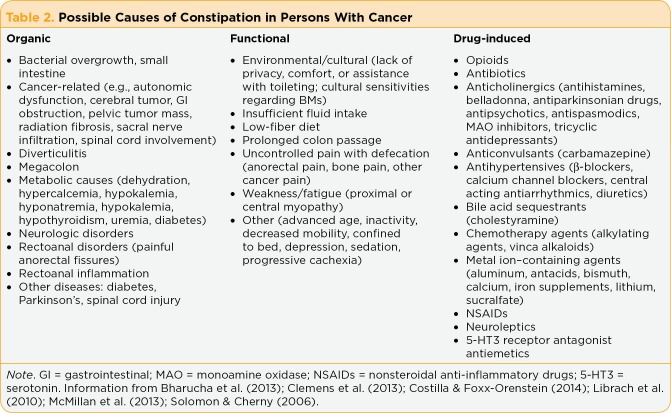
Possible Causes of Constipation in Persons With Cancer

It is prudent to anticipate opioid-induced constipation (OIC) in patients starting or taking opioid analgesics and to start prophylactic management. Opioid receptors are expressed throughout the enteric nervous system (ENS) in the gastrointestinal (GI) tract, and OIC occurs because opioids bind to ENS receptors and induce delayed gastric emptying, decreased intestinal secretion, slowed propulsive contractions, decreased colonic motility, increased fluid absorption from stool, and increased sphincter tone, which result in retention of hard, dry stools (McMillan et al., 2013; Mori et al., 2013). Tolerance to OIC rarely develops, and patients may skip or decrease opioid doses or stop taking their opioid altogether to relieve OIC. This problem leads to increased pain, reduced activities of daily living, and reduced quality of life (Camilleri et al., 2014).

## FECAL IMPACTION

Chronic, unmanaged constipation can progress to fecal impaction, which further impairs patients’ quality of life and increases health-care costs (Hussain et al., 2014). Feces remaining in the colon for longer than normal cause great water and salt resorption from the colon, which further slows peristalsis and stool packing. In low impactions, stool accumulates in the descending colon to the rectum, and in high impactions, stool fills the ascending colon (Bisanz, 2007). A hard, dry fecal mass essentially obstructs the colon or rectal vault and may be accompanied by overflow incontinence as diarrhea seeps around the stool mass (Solomon & Cherny, 2006).

## FOCUSED BOWEL HISTORY AND PHYSICAL ASSESSMENT

A patient’s self-report of constipation, which can be gained by a reliable and valid screening tool (see "Assessment of Constipation in Patients With Cancer" in the May/June 2016 issue of JADPRO), should be incorporated into a more thorough focused history and physical examination to confirm constipation (and rule out bowel obstruction; Librach et al., 2010; Selby & Corte, 2010). Assessment parameters include:

- What the patient considers "normal" BMs
- Usual bowel habit, duration of feeling constipated, date of last BM
- Current stool appearance (consistency, color, odor, blood, mucous)
- Associated symptoms (e.g., nausea, abdominal fullness, bloating, gas, diarrhea, tenesmus)
- Likely causes and contributing factors (see Table 2):
  - Medication history, including laxatives, suppositories, enemas
  - Medical conditions affecting laxative selection (e.g., vocal cord paralysis, which precludes mineral oil, or impaired renal function, which contraindicates magnesium salts)
- Current diet and desire to eat as well as fiber intake (can patient consume fiber to 30 G per day and drink sufficient fluids to maximize bulk effects and avoid exacerbating constipation?)
- Activity level, altered mobility, fatigue, or weakness, which may interfere with usual normal BMs.

Laboratory tests, per se, are not indicated except to identify contributing factors for constipation (e.g., hypercalcemia or diabetes) or risks from particular interventions (e.g., blood urea nitrogen and creatinine levels to assess renal function, and white blood cell and platelet counts to identify risks with rectal administration or manual disimpaction). Similarly, a flat plate of the abdomen may differentiate severe constipation, fecal impaction, and obstruction (Bisanz, 2007).

The physical examination focuses on the patient’s abdomen and rectum. If the abdomen appears distended, look for visible peristalsis. Auscultation will distinguish among normal, hyperactive, and absent bowel sounds. Palpable masses—particularly left-sided (descending colon)—must be examined by deep palpation to distinguish stool (which indent) from tumors (which do not; Clemens et al., 2013; Librach et al., 2010). A fecal mass with gas trapped in the bowel may feel like crepitus, and percussion may differentiate ascites and a gas-filled bowel. A tympanic, distended abdomen with mild diffuse tenderness may signal fecal impaction (Hussain et al., 2014).

It is important to consider factors that contraindicate rectal examination (e.g., neutropenia or thrombocytopenia), cultural sensitivities, and ensuring privacy during the examination. Poor internal anal sphincter tone may indicate spinal impingement or compression (ask the patient to strain or push down while doing the exam to evaluate). Patients who have sharp, knifelike pain during the examination may have mucosal injury (Costilla & Foxx-Orenstein, 2014). A dilated rectum or no palpable stool in the rectal vault may indicate higher constipation in the sigmoid colon (Hussain et al., 2014; Librach et al., 2010). Hard, dry stool in the rectum with fecal impaction directs the first management step: elimination of impacted stool before starting oral laxative therapy (Clemens et al., 2013).

## MANAGEMENT

Managing constipation aims to alleviate patient discomfort, restore and maintain satisfactory and comfortable BMs, prevent related symptoms of constipation or laxatives (e.g., nausea, bloating, and abdominal pain), improve a patient’s sense of control of bowel habits, and preserve comfort and dignity (Clemens et al., 2013; Larkin et al., 2008; Librach et al., 2010). Interventions are somewhat based on prognosis and how distressing constipation is to the patient. Management approaches may include nondrug, adjunct measures but center on pharmacologic interventions.

**Nondrug Measures**

There is meager evidence for lifestyle modifications (e.g., ensuring patient privacy and comfort, recommending the patient try to defecate the same time each morning or after eating) and dietary fiber (Andrews & Morgan, 2013; Foxx-Orenstein, McNally, & Odunsi, 2008) for patients with cancer. Increasing oral fluids and exercise may not be useful (or possible). Fiber has limited benefit and cannot prevent or treat OIC, which requires prophylactic laxatives (Clemens et al., 2013; Wald, 2007). Similarly, suggesting a fiber supplement to an anorexic and mildly dehydrated patient with advanced disease is counterproductive, because fiber can worsen early satiety and requires drinking plenty of fluids to be effective (Larkin et al., 2008). The results of a meta-analysis of five studies that examined the effect of dietary fiber on constipation concluded fiber intake significantly increased the number of BMs but did not improve stool consistency, laxative use, or painful BMs (Yang, Wang, Zhou, & Xu, 2012). These authors suggested dietary fiber might be effective for mild to moderate, but not severe, constipation. Relatively healthy patients with a good prognosis can find recipes for homemade fiber supplements (for examples, see http://www.in.gov/fssa/files/Bowel_Aid_Food_Recipes_OR-FM-HS-CN-12(11-6-09).pdf).

Observational studies, case reports, and clinical reviews suggest abdominal massage may be another helpful adjuvant measure for constipation in palliative care patients, elderly individuals, patients with spinal cord injury, or those with postoperative ileus (Sinclair, 2011). There is evidence of the physiologic effects of abdominal massage to increase GI motility and digestive secretions, relax sphincters, shorten GI transit time, decrease abdominal discomfort, and enhance rectal loading, which increases the sensation of having to have a BM (Andrews & Morgan, 2013; Lamas, Lindholm, Stenlund, Engstrom, & Jacobsson, 2009). One prospective study found abdominal massage was not immediately effective, but after 8 weeks, patients in the massage group had significant reductions in GI symptoms and abdominal discomfort and increased BMs vs. the control group (Lamas et al., 2009). The nurse investigators concluded the delayed effect of abdominal massage complements laxatives. Clinicians can teach abdominal massage to patients or caregivers, which enhances patients’ self-management and relaxation (Andrews & Morgan, 2013). Many websites clearly and succinctly explain the procedure, and most have helpful illustrations (for examples, see https://www.youtube.com/watch?v=N39GIWquhWg or http://www.guysandstthomas.nhs.uk/resources/patient-information/gi/abdominal-massage-for-constipation.pdf)

**Pharmacologic Therapy**

Pharmacologic agents for constipation include oral, over-the-counter (OTC) laxative products, rectal suppositories and enemas, and methyl-naltrexone (a prescription parenteral drug; see Table 3). Oral products are classified as bulking agents, stool softeners, stimulant laxatives, and osmotic laxatives. There are few randomized controlled laxative studies in cancer or palliative care, and laxative selection is largely based on clinical experience and expert consensus recommendations. All laxatives and methylnaltrexone are contraindicated in patients with suspected bowel obstruction (Woolery et al., 2008).

**Table 3:**
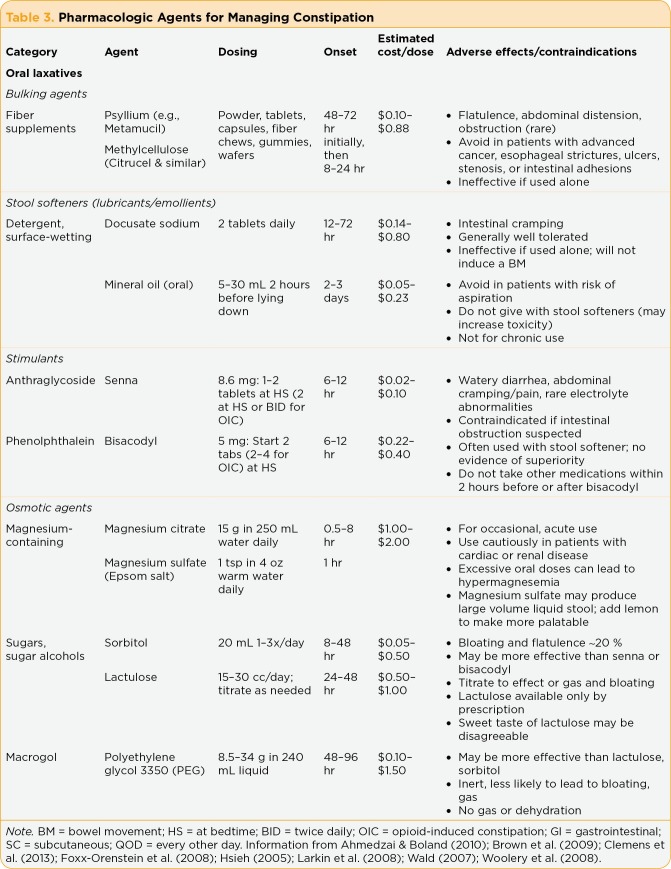
Pharmacologic Agents for Managing Constipation

**Table 4:**
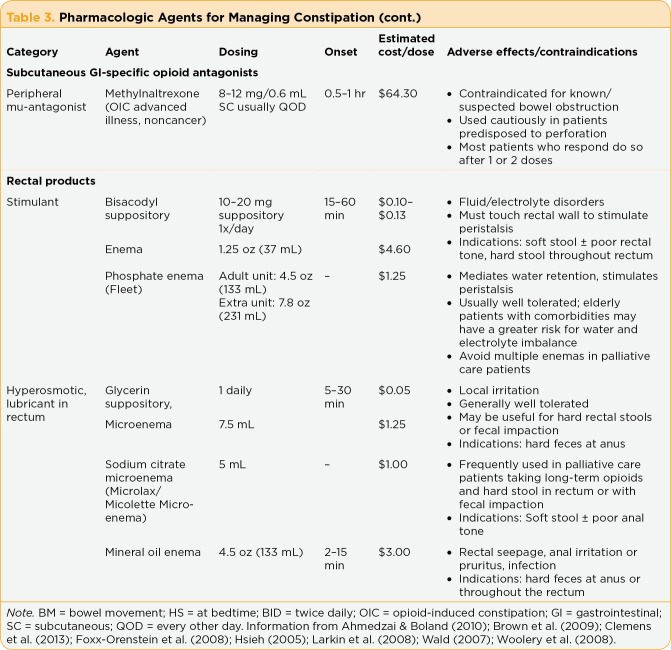
Pharmacologic Agents for Managing Constipation (cont.)

*Bulking Agents*: Soluble (e.g., psyllium, pectin) and insoluble (methylcellulose) fiber products induce a stretch reflex in the intestinal wall, which increases propulsive activity, water absorption, and bacterial proliferation in the colon, leading to softer and larger stool masses and easier BMs (Candy et al., 2015; Costilla & Foxx-Orenstein, 2014; Larkin et al., 2008; Wald, 2007). Bulk laxatives are not effective for already-constipated cancer patients, especially those taking opioid analgesics or anticholinergic drugs. They are most appropriate for patients who do not consume adequate dietary fiber, have a good performance status, are experiencing mild to moderate constipation, and have normal GI transit.

Bulking laxatives are generally well tolerated, but side effects may include bloating and excessive gas. Bulking laxatives may worsen symptoms in patients with slow-transit constipation caused by opioids or anticholinergic agents or with anorectal dysfunction. In addition, bulking laxatives are not recommended for patients with advanced disease who may not drink sufficient fluids to avoid intestinal obstruction or fecal impaction (Candy et al., 2015; Woolery et al., 2008). Rare adverse effects of bulking laxatives include esophageal obstruction and psyllium hypersensitivity (Xing & Soffer, 2001). Acute esophageal obstruction after ingesting a bulking laxative has occurred in patients with or without mild dysphagia. The actual incidence of hypersensitivity is not known, but 5% of individuals preparing psyllium experienced shortness of breath, wheezing, or hives within 30 minutes after preparing psyllium laxatives. Bulking laxatives can also significantly reduce feelings of hunger, increase a sense of satiety, and delay gastric emptying—all negative effects in patients with advanced cancer.

*Stool Softeners (Lubricants or Emollients)*: Docusate (Colace, Surfak) and mineral oil (liquid paraffin) are stool softeners that act as detergents (surface-wetting agents) and allow water to enter the bowel and lower surface tension and as lubricants/emollients to soften and lubricate stools (Costilla & Foxx-Orenstein, 2014; Hsieh, 2005; Pitlick & Fritz, 2013). Used alone, stool softeners are less effective than psyllium and are ineffective for constipated individuals. Patients must increase fluid intake with these agents to soften stools; this may be useful for patients with hemorrhoids or anal fissures, which cause painful defecation, and for those who should otherwise avoid straining (Candy et al., 2015; Woolery et al., 2008). However, docusate would be contraindicated in patients with poor oral intake who cannot increase fluids or in those with overly dry stools secondary to prolonged time in the colon secondary to OIC. Docusate may enhance gastrointestinal or hepatic uptake of other drugs, but the magnitude of this effect and its clinical significance in altering hepatotoxicity are unknown (Xing & Soffer, 2001).

It has also been proposed that regular use of mineral oil might impede absorption of fat-soluble vitamins, but this has not been confirmed. Mineral oil poses a risk for aspiration pneumonia in patients with swallowing disorders and can cause perianal irritation because of seepage of oily material (Xing & Soffer, 2001).

Some palliative care and hospice clinicians are familiar with oral petroleum jelly (OPJ), also called "Vaseline balls," as an alternative to mineral oil used after unsuccessful treatment of constipation with standard laxatives. Tavares, Kimbrel, Protus, and Grauer (2014) did an online survey including a convenience sample of 353 physicians, nurse practitioners, nurses, and pharmacists (67% of whom were familiar with OPJs), which was used in approximately 10% of patients. Most of the clinicians (87%) rated OPJs as effective or very effective in inducing BMs within 24 hours of administration.

Oral petroleum jelly is made by chilling petroleum jelly, forming it into pea- to marble-sized balls, rolling it in powdered or granulated coatings to enhance palatability, and freezing or refrigerating it until use. Freezing hypothetically makes OPJs safer than mineral oil, because they do not liquefy until they reach 100.4°F in the GI tract. At that point, they are thought to act like mineral oil to coat and soften feces causing high impaction. There is no agreed-upon dosing size or interval for OPJ.

*Stimulants*: Stimulant laxatives include senna (Senokot, Ex-Lax), bisacodyl (Dulcolax, Correctol), and castor oil. They induce a strong laxative effect by directly stimulating submucosal and deeper myenteric plexuses in the bowel wall to cause forceful peristalsis, and increased water and electrolytes release into the intestine (Costilla & Foxx-Orenstein, 2014; Hsieh, 2005; Larkin et al., 2008; Wald, 2007). Senna must be administered orally to be metabolized and activated in the GI tract, whereas bisacodyl can be given orally or by suppository, as it is activated by intestinal glucuronidase. Stimulant laxatives are considered first-line options and are often used for OIC, especially senna, which counters opioid-induced–segmenting activity and is the least expensive (Pitlick & Fritz, 2013; Twycross et al., 2012; Woolery et al., 2008).

*Osmotic Agents*: Nonabsorbable sugars and polyethylene glycol (PEG) without electrolytes are osmotic laxatives—first-line drugs because of their rapid onset, low number of adverse effects, ease of use, and relatively low cost. Polyethylene glycol is an excellent choice because of its softening and stimulating effects (Pitlick & Fritz, 2013). These poorly absorbed ions or molecules cause an osmotic gradient within the small intestine and lead to water retention, faster intestinal transit, and softer feces (Clemens et al., 2013; Costilla & Foxx-Orenstein, 2014; Hsieh, 2005; Twycross et al., 2012; Wald, 2007).

Magnesium salts (milk of magnesia, magnesium sulfate [Epsom salts], and magnesium citrate) are also osmotic. However, the ions of magnesium-containing cathartics are partially absorbable, so serious adverse effects related primarily to excessive ion absorption may cause metabolic disturbances (Xing & Soffer, 2001). Repetitive dosing can lead to hypermagnesemia and symptoms of hyporeflexia and lethargy, which can progress to a medical emergency with hypotension, shock, prolonged QT interval, respiratory depression, and even death. Magnesium laxatives should be used for acute evacuation (to rapidly induce a BM) and avoided in patients with renal insufficiency. However, hypermagnesemia has occurred in patients with normal renal function. Chronic use of these agents may also exacerbate fluid overload in patients with congestive heart failure.

Lactulose and sorbitol are indigestible and nonabsorbable sugars, which colonic bacteria metabolize into compounds that increase stool acidity and osmolality, causing fluid to be drawn into the colon and peristalsis to increase (Hsieh, 2005; Wald, 2007). Bacterial fermentation with lactulose also causes gas production, abdominal cramping, and flatulence—especially with larger doses. On the other hand, colonic bacteria cannot degrade PEG (MiraLAX), which is therefore less likely to cause bloating and gas. Once-daily PEG usually induces laxation, and there is some evidence it is superior to lactulose for chronic constipation (Solomon & Cherny, 2006; Woolery et al., 2008). Potential electrolyte imbalances that can occur with osmotic laxatives including lactulose or sorbitol include hypernatremia and hypokalemia (Xing & Soffer, 2001). These events occur because more water than sodium stays in the GI tract, and potassium can be lost in loose stools.

*Peripheral Opioid Antagonists*: Methylnaltrexone is the only peripheral mu-opioid antagonist approved for OIC in patients with advanced illness or non–cancer-related pain. Peripheral opioid antagonists are not laxatives, per se. As discussed, opioids not only bind to central nervous system opioid receptors, but to mu receptors in the ENS to ultimately cause OIC (Chey et al., 2014; Wald, 2016). Methylnaltrexone and other ENS antagonists (e.g., naloxegol and alvimopan) competitively bind to GI opioid receptors and antagonize ENS effects, but they cannot cross the blood-brain barrier to decrease analgesia. Methylnaltrexone does not replace the need for laxatives for constipation from other causes or other manifestations such as abdominal cramping and delayed gastric emptying (Ahmedzai & Boland, 2010).

Most adults who have OIC (despite receiving laxatives) have a BM within 4 hours of receiving subcutaneous (SC) methylnaltrexone (Portenoy et al., 2008). The most common adverse effects of methylnaltrexone are mild abdominal pain, diarrhea, nausea, rectal gas, or vomiting. It is initially given every other day in doses based on a patient’s weight. Dosing intervals may be extended or reduced, but methylnaltrexone should not be given more than once a day. Severe renal impairment (creatinine clearance < 30 mL/min) requires a 50% dose decrease (Pitlick & Fritz, 2013). Because of its high cost compared with other oral and rectal laxatives, methylnaltrexone would be justifiable only after optimal doses of other laxatives have been ineffective (Argoff et al., 2015; Twycross et al., 2012).

*Rectally Administered Suppositories and Enemas*: Rectal laxatives—suppositories or enemas—are generally safe and effective and are a preferred option when rapid and predictable evacuation of stool from the rectum and distal colon is desirable, such as in patients with fecal impaction, complete spinal cord injury, or neurogenic bowel (Brown, Henderson, & McDonagh, 2009; Woolery et al., 2008). If a patient has fecal impaction, management may include disimpaction, evacuation of the colon, and a maintenance bowel regimen to prevent recurrence (Hussain et al., 2014). In patients with cancer, the first step would be an enema or suppository to soften or lubricate the stool in the rectum and distal colon to allow for easier passage.

On the other hand, manual disimpaction (with light sedation) would be a last choice for cancer patients because of patient discomfort, possible embarrassment, and risk for complications (Hussain et al., 2014; Solomon & Cherny, 2006). Before manual disimpaction, the clinician must rule out contraindications—especially neutropenia and thrombocytopenia—and consider the patient’s relative risks for iatrogenic mucosal injury or perforation, syncope, or arrhythmia related to vagal stimulation (Hussain et al., 2014). A prophylactic daily oral laxative regimen should be given with or shortly after rectal medications have relieved the impaction (Brown et al., 2009; Solomon & Cherny, 2006).

There is no evidence to recommend one type of product over another, but microenemas are preferred over phosphate enemas, because they have smaller volumes and fewer adverse effects and are similarly effective (Brown et al., 2009). Tap-water enema and glycerin suppositories are also good choices because they usually induce BMs in 30 to 60 minutes and have few side effects, although rectal administration may cause mild rectal irritation (Pitlick & Fritz, 2013; Solomon & Cherny, 2006).

Bisanz (2007) recommends a mineral oil enema as the first step for patients with low or high impactions and a second enema (e.g., soap and tap water ≤ 1 L) 1 hour later if needed. A patient’s general health and comorbid conditions dictate the amount of enema fluid tolerated. If the patient lies on his or her right side with the enema tube in place in the rectum for 20 minutes, he or she may be able to hold the enema fluid. Removing the enema tube usually causes the immediate urge to defecate. Large meals and hot liquids before enemas or disimpaction increase peristalsis and abdominal colic and should be avoided (Woolery et al., 2008). If the patient does not experience liquid stool and is not nauseated after the first or second enema, magnesium citrate or PEG is a first-line choice. Lactulose or sorbitol (30 mL four times per day) is another option but is more likely to cause gas, bloating, and abdominal cramps. Any of these enemas can be repeated in 12 hours if needed.

Sodium (Fleet) phosphate enemas are commonly used in palliative care and are considered relatively safe. However, individuals older than 65 years and others with comorbidities may be at greater risk for water and electrolyte abnormalities (Ahmedzai & Boland, 2010). There are reports of sodium phosphate enemas causing significant morbidity and mortality in elderly patients or those with renal insufficiency, even when standard doses are given (Ori et al., 2012; Xing & Soffer, 2001). Affected patients typically present within 24 hours (although this may occur up to 72 hours later) with acute and life-threatening hyperphosphatemia and reciprocal hypocalcemia, nausea and vomiting, metabolic acidosis, acute renal failure, and perhaps hypernatremia and hypokalemia. This is a medical emergency, and patients require fluid resuscitation and sometimes hemodialysis.

The pathogenesis of extreme hyperphosphatemia is linearly related to enema retention time; when a stool is not expelled within a short time, phosphate is absorbed from the colon into the circulatory system. Sodium phosphate enemas should thus be avoided in patients with fecal impaction, paralytic ileus, or bowel obstruction, as well as in patients with fluid-electrolyte disturbances. Thus, if the patient does not expel enema stool within 30 minutes, other measures must be taken to evacuate the bowel to minimize absorption of phosphate.

## PUTTING IT ALL TOGETHER

Advanced practitioners (APs) can be instrumental in developing and implementing bowel protocols in their practice settings. These protocols are not only appropriate for patients undergoing cancer treatment, but often become useful for cancer survivors and those who experience progressive disease and receive palliative care. As discussed, there is little research to support laxative selection and dose escalation for patients with cancer, OIC, or those receiving palliative care; constipation management recommendations are largely based on consensus (Brick, 2013; Camilleri et al., 2014; Pitlick & Fritz, 2013). Such recommendations have been published by Canadian (Librach et al., 2010) and European (Larkin et al., 2008) authors. In the United States, the Oncology Nursing Society also has links to clinically useful resources (https://www.ons.org/practice-resources/pep/constipation) and summary recommendations (Woolery et al., 2008).

Factors to considers when formulating a laxative plan include the patient’s prognosis and relative health, whether the patient is already constipated or constipation is likely (e.g., the patient is starting opioid therapy for pain control, whether they have other risk factors for constipation or are taking more than one drug with anticholinergic properties), planning strategies that may help the patient adhere to a laxative plan (e.g., calendars, pill boxes, pill reminder apps), and making timely adjustments to the plan as indicated.

Relatively healthy patients who are not constipated or have mild constipation, and are not taking opioid analgesics, can be advised to increase dietary fiber (or use bulking laxatives or fruit pastes), fluids, and exercise, which may be helpful to prevent or minimize constipation. Conversely, these actions may actually be harmful to patients with progressive or advanced disease. Similarly, bulking laxatives and stool softeners have little (if any) effect on chronic constipation and at best should be considered adjuvants to other laxatives (Hawley & Byeon, 2008). Although many clinicians advise patients to take senna plus docusate (e.g., Senokot S), particularly for OIC, there is no evidence that docusate adds any benefit (Ahmedzai & Boland, 2010; Hawley & Byeon, 2008). Furthermore, senna plus docusate may increase the pill load for patients who take generic products and does not decrease abdominal side effects.

Senna, lactulose, and PEG are similarly effective first-line laxatives (Ahmedzai & Boland, 2010; Candy et al., 2015; Hawley & Byeon, 2008; Wald, 2016). Cost can initially guide laxative selection. Senna is the least expensive and probably most widely used, and it can be started as a single agent. Patients with OIC generally need higher doses than patients who are constipated secondary to other causes, but there is no direct and predictable relationship between increasing doses of opioids and higher doses of laxatives. As can be seen in the Figure, a proposed strategy for starting senna is one dose at bedtime or perhaps two doses per day (each morning and at bedtime) for a patient who has OIC (Ahmedzai & Boland, 2010; Hawley & Byeon, 2008; Twycross et al., 2012). If senna is not effective or tolerable, it is not unreasonable to try bisacodyl. Ultimately, the patient may take two or more laxatives, and they should be from different categories, such as a stimulant laxative and an osmotic laxative (Wald, 2016).

**Figure 1:**
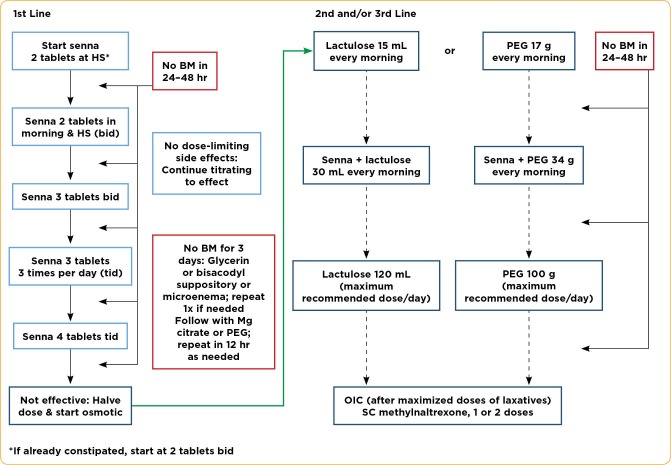
Constipation Management Algorithm. First-line recommendations for laxatives are to start with a stimulant, usually senna, two tablets at bedtime (HS), and to increase in a stepwise fashion. Patient who have opioid-induced constipation (OIC) generally require larger doses and may be started at two tablets twice daily (bid). Senna is similarly effective as lactulose and polyethylene glycol (PEG), but they are second- or third-line choices and are given in the morning because of a faster onset of action. Laxatives may need to be changed because of abdominal cramping and gas. Patients may need more than one laxative, so drugs with different mechanisms should be used. Methylnaltrexone is reserved for patients who have OIC and after other maximized doses of oral and rectal laxatives. BM = bowel movement; Mg = magnesium; SC = subcutaneous; tid = three times a day. Information from Ahmedzai & Boland (2010); Hawley & Byeon (2008); Pitlick & Fritz (2013); Selby & Corte (2010); Twycross et al. (2012); Wald (2016).

Advanced practitioners must work closely with the constipated individual with cancer to find the best regimen for him or her, and should give the patient written instructions to buy generic single or combined products that are inexpensive, as the same branded products are expensive. Initial instructions are to take a dose at bedtime, add a morning dose as needed, and then titrate as necessary or change to an alternative product. Some patients with OIC benefit from adding an osmotic laxative (lactulose [Enulose] or polyethylene glycol [MiraLAX]) or intermittent magnesium citrate, which should be taken early in the day. These agents act more rapidly than stimulant laxatives but may cause gas and bloating, so enemas (oil retention, then Fleet) may be another option for constipated patients. On the other hand, bulk-forming laxatives (bran, psyllium, calcium polycarbophil, and methylcellulose) are contraindicated for patients with advanced illness, a poor functional status, or low oral intake of food or fluids. Use of these agents will lead to increased constipation, possible fecal impaction, and anorexia (Clemens et al., 2013). Subcutaneous methylnaltrexone, which antagonizes opioids bound to peripheral mu receptors in the GI tract and does not cross the blood-brain barrier, every other day is an alternative when standard laxatives have not been effective (Argoff et al., 2015; Wald, 2016).

## CONCLUSIONS

Constipation is a high-frequency, high-impact problem for individuals with cancer. Advanced practitioners have important roles in recognizing those at risk, screening for constipation and impaction, and developing logical implementation plans that center on oral laxatives. Close patient follow-up is crucial to determine optimal doses that alleviate patient symptoms without being overly burdensome without causing distressing abdominal adverse effects—which can occur with any laxative. As new evidence-based data become available, APs can also share this information and collaborate with physician and nurse colleagues.
